# The Long Journey of Extracellular Vesicles towards Global Scientific Acclamation

**DOI:** 10.34172/apb.2023.049

**Published:** 2022-07-02

**Authors:** Marco Pirisinu

**Affiliations:** ^1^Department of Biomedical Sciences, College of Veterinary Medicine and Life Sciences, City, University of Hong Kong, Hong Kong.; ^2^Jotbody HK Limited, New Territories, Hong Kong.

**Keywords:** Extracellular vesicles, Therapeutic applications, Biotechnology market, Clinical trials

## Abstract

Extracellular vesicles (EVs) are a heterogeneous class of cell-derived vesicles that are responsible for eliciting a wide array of biological processes. After decades of intense investigation, the therapeutic potential of EVs will be finally explored in a series of upcoming clinical trials. EVs are rapidly changing the understanding of human physiology and will undoubtedly transform the field of medicine. The applicability of EVs as diagnostic biomarkers and treatment vectors has captured the attention of the scientific community and investors, facilitating the rapid progression of numerous EVs-based platforms. This mini-review provides an outline of the pioneering discoveries, and their respective significances, on progressing EVs toward clinical use. We focus the attention of the readers on several promising classes of EVs that hold major opportunities to translate in clinical practice. Market analysis and future challenges facing EVs-based therapies are also discussed.

## Introduction

**Figure 1:**
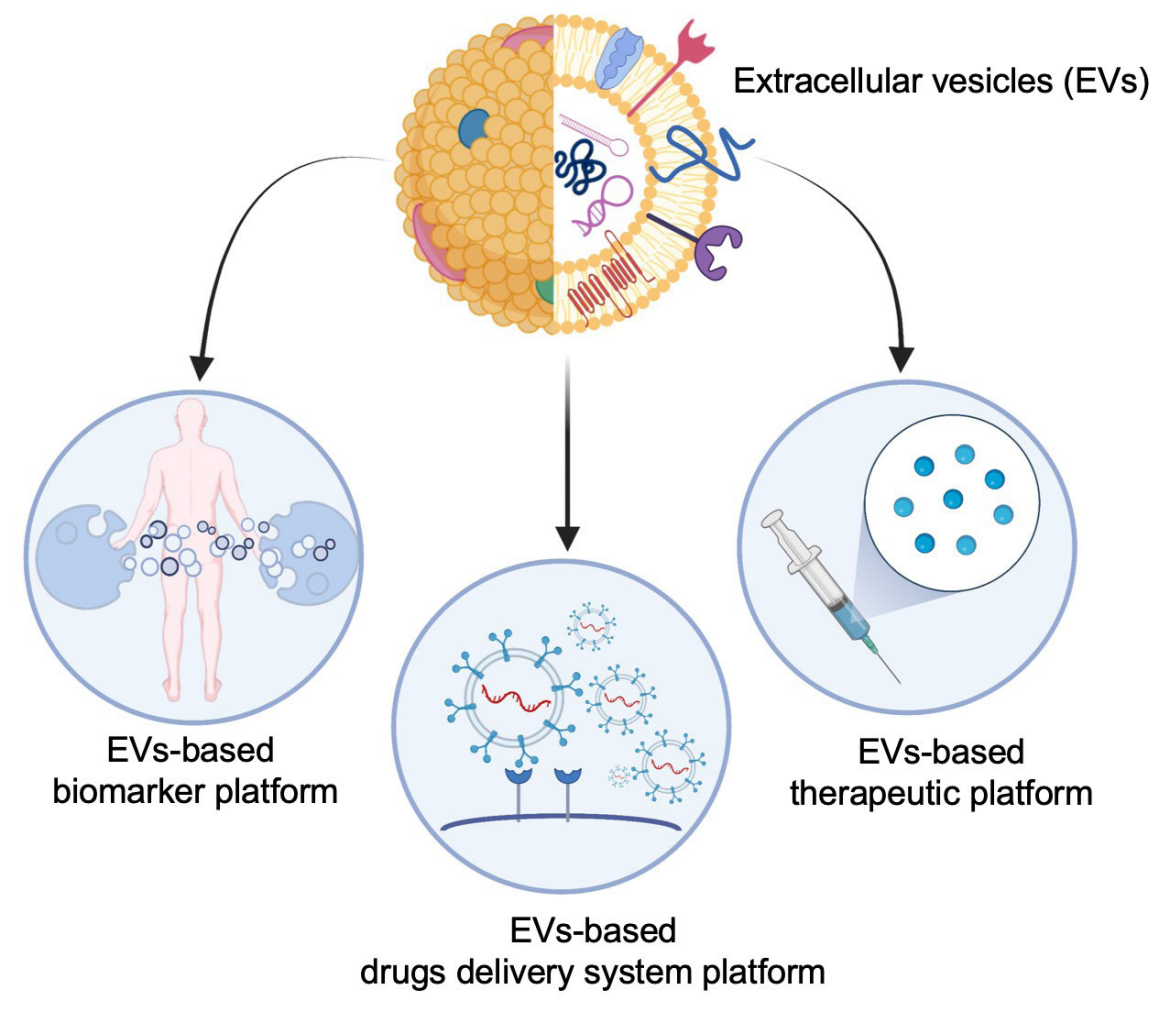
Extracellular vesicles (EVs) based therapeutic platforms: intrinsic natural properties allow EV to be easily harnessed from different biological fluids and adopted for monitoring the patient treatment response (EVs-based biomarker platform). Moreover, the mirror properties of EVs has been harnessed to develop EVs-based therapeutic platform which includes mesenchimal stem cells derived EVs, mostly applied in the area of regenerative medicine, and dendritic cells derived EVs for immunotherapeutic application. The majority of EVs finds application as carrier for different drugs including small synthetic molecules, natural products and oligonucleotide therapeutics (EVs-based drugs delivery platform)

 Extracellular vesicles (EVs) are cell-derived nano-sized particles that travel throughout an organism to deliver proteins, RNAs, and DNAs to recipient cells. This revolutionary concept has triggered immense excitement from the global biomedical research community. Although a modest amount of research illuminating the nuanced aspects of EVs biology and its impact on disease progression has been produced,^1^ the vast majority of the preclinical studies have primarily focused on the therapeutic application of EVs, such as real-time biomarker monitoring or drug delivery systems (Figure 1).^2,3^

 Explosive interest is growing around the EVs field as their biological relevance is becoming increasingly recognised.^4^ As such, the enormous medical potential of EVs has prompted a fast action from investors and the biomedical industry. An increasing number of pharmaceutical companies are pivoting their financial investment into R&D and commercialization of naïve or functionalized EVs-based platforms.^5^ Additionally, smaller biotech companies and academic laboratories are attempting to utilize the properties of EVs to develop innovative strategies that will accelerate the development of next generation EVs-based therapies.^6^

 EVs have high therapeutic potential and have yielded unexpected results over the last decade. Nearly 80% of the articles pertaining to EVs listed on PubMed were published within the past five years. Similarly, the number of clinical trials utilising EVs has increased seven fold.^7^

 However, despite a hearty reception, the limited understanding of EVs biology is hampering the progression of EVs toward the clinic. In this respect, EVs biotechnology currently remains a promising avenue with few suitable candidates for successful clinical translation.

 This mini-review analyses the evolution of the EVs field and focuses on the findings of pioneering studies that have influenced the current trajectory of EVs-based therapeutics; market, trends, drivers, and major players in the EVs research field are also critically examined. Specific subsections describe recent progresses of EVs as delivery vectors and the current status of EVs classes that hold the largest opportunity for transitioning from bench to bedside. Finally, a critical discussion explores the future direction and technical obstacles that research may encounter on the road to successful implementation of EVs-based therapy.

## EVs classification and definition

 EVs are considered a cellular secretome and have been observed in a variety of plant and animal species.^8–13^

 To date, the scientific literature has documented a myriad of cell-released vesicles with differing sizes, similar functions, and unclear biogenesis. Diverse parameters have been gradually proposed to distinguish EVs of different subtypes, such as microvesicles or exosomes. Microvesicles (100 nm - 2 µm), bud directly from the cell membrane showing an irregular shape whereas exosomes (30 nm - 200 nm), originate through the endosomal dependent pathway and are more regularly shaped.^14,15^ However, the overlapping size of vesicles belonging to both classes and the absence of standardized methods to study EVs biogenesis reveal our limited knowledge and the inappropriateness of such nomenclature.^16^ Thus, it is recommended to distinguish the EVs according to their physical and biochemical properties.

 The analysis of EVs content is undoubtedly the more reliable method to distinguish between “microvesicles” and “exosome”. Due to the nature of their biogenesis, exosomes contain a variety of lipids, proteins and nucleic acids including mitochondrial DNAs which are absent in microvesicles.^17^ More importantly, exosome can be identified via the presentation of “exosomal marker proteins” – Alix, TSG101, HSC70, CD63, CD9, CD81 and HSP90β among others – which characterize exosomes independently of cell origin.^18^ Surface markers associated with microvesicles include integrin-β, CD40, selectins, heat shock proteins and post translational modifications, including glycosylation and phosphorylation;^19,20^ however, it is worth of mentioning that the proteomic profile of microvesicles is significantly impacted by downstream isolation and purification methods.^16^

 Every cells of the human body generates EVs, herein the identification of specific markers is a well-corroborated approach to validate their origin. In this respect, ACTN4 and CD11c are canonical markers for dendritic cells derived EVs^21^; CD235a (GPA) serves as a marker for red blood cells derived EVs^22^; CD31 and CD73 are presented on the surface of platelets and mesenchymal stem cells derived EVs respectively.^23,24^ In addition, EVs can be isolated from any biological fluid but the lack of standardized protocols has resulted in conflicting results between different studies. Indeed, the use of a variety of methods for isolation and purification significantly impacts the size, content, shape, and purity of isolated EVs.^25^ In this regard, as suggested by the minimal information for studies of EVs (MISEV), a satisfactory EVs characterization should provide both lipid-bound extracellular protein and cytosolic proteins to confirm the presence and origin of EVs.^26^

 In summary, although some research groups are in favour of this classification scheme, the umbrella term “extracellular vesicles” has been accepted to refer to the entire heterogeneous group of vesicles released by both eukaryotic and prokaryotic cells; and the source of origin is usually specified (e.g. platelet-derived EVs). Accordingly, in this review the term “EVs” refers to any cell derived vesicles, except as otherwise provided.

## Understanding the impact of pioneering studies on the actual success of EVs

 Our current understanding of EVs is built upon the foundation of over 70 years of intense research. Historically, EVs were first documented in the 1940’s by Chargaff et al during their investigation of the biological composition and significance of blood-derived thromboplastic protein.^27^ Two decades later, the intriguing term “platelet dust” was used to describe the lipid-containing particles enriched in fibrinogen that likely originated from platelets.^28^ These early studies by Chargaff and many others created the basis for a new field of investigation that is now known as membrane trafficking. However, at that time, the origin, biogenesis, and function of EVs were unclear and they were considered as inert cellular waste.

 In 1981, convincing data demonstrated that nano-sized vesicles were released during the *in vitro* culture of normal and neoplastic cells.^29^ In addition, the same authors can be credited for coining the term “exosome” to refer to exfoliated plasma membrane fragments (40 nm in diameter).

 In the 1970s, endocytosis and receptor recycling mechanisms were the most prevalent research topics. In lieu with the trend, several research groups began investigating the transferrin receptor, which is enriched in reticulocytes but almost totally lost during their maturation.^30^ In 1983, interest on the fate of transferring receptor and the development of innovative techniques – such as the use of gold or radiolabelled particles, ultracentrifugation, and electron microscopy - lead to the isolation, visualization, and mapping of the transferrin receptor on reticulocyte-derived vesicles.^31,32^ Rat and sheep reticulocytes were subsequently used to further investigate the cellular origin of EVs.^33,34^ The similarity in lipidic composition, and in particular the abundance in sphingomyelin, undoubtedly proved that those vesicles were of reticulocyte origin. Moreover, as reticulocytes are non-nucleated, these findings indirectly suggested that nuclear DNA was dispensable for EVs biogenesis and that EVs were therefore incapable of self-replication. Several years later, a fairly comprehensive study demonstrated that sheep reticulocyte-derived EVs inherited additional plasma membrane proteins, including the nucleoside transporter and acetylcholinesterase.^35^

 Despite the magnitude of those discoveries, the scientific community were unaware of the functional role of EVs. Interest subsequently waned, with only a small number of labs continuing their investigations on EVs biology.

 In 1996, over a decade later, interest exploded again due to a cornerstone paper that brought remarkable insights into the biological function of EVs. The research article convincingly showed that B lymphocytes, professional antigen-presenting cells, released nano-sized vesicles capable of modulating specific T cell responses *in vitro.* Importantly, their findings also implied that EVs can be involved in antigen presentation *in vivo.*^36^

 Without a doubt, the work of Raposo et al generated an increased awareness of the role of EVs in cell-to-cell information transfer. As a result, interest in EVs was revitalised, with an enormous number of scientific papers investigating the role of EVs in different cellular activities produced in the last 25 years.

 Due to Raposo and others, EVs are currently recognised as the ultimate mediators of intercellular signalling in physiological and pathological conditions. Immune modulation,^37^ thrombosis,^38^ vascular dysfunction,^39^ *de novo* mineralization,^40^ alteration of the tumor microenvironment,^41^ and progression of viral infection^42^ are just a few of the biological activities regulated via EVs.

 The results of the aforementioned studies were major breakthroughs in EVs research. The research findings demonstrated that the EVs were dedicated to mirror the biology of their parental and also paved the way for developing three of the currently most promising EVs based platforms: mesenchymal stem cell-derived EVs (MSCEVs), red blood cell-derived EVs (RBCEVs), and dendritic cell-derived EVs (DExs). The therapeutic applications of these EVs based platforms will be discussed in the following sections.

 A renewed spike in publications appeared over the course of the 2006/2007 year. Within a short span, several key articles demonstrated that oligonucleotides could be horizontally transferred between cells via EVs while retaining their biological activity in the new location.^43,44^ More importantly, the authors highlighted that EVs can sequester and transport RNAs that would otherwise be enzymatically degraded.

 Currently, RNA-based drugs possess incredible prophylactic potential and are at the forefront of personalized medicine.^45^ However, several factors such as stability, immunogenicity, and lack of efficient *in vivo* delivery vehicles has limited the development of mRNA-based therapeutics. The application of EVs as carriers of drug molecules, particularly oligonucleotide therapeutics, has proceeded relatively straightforward and offers more promise than previously explored competitive technologies. This finding has enormous implications for human health and has encouraged the development of many EVs platforms specifically tailored for the delivery of oligonucleotide therapeutics.^46-49^

 The interest in the biological aspects and therapeutic application of EVs research has reached new heights, with hundreds of scientific papers published yearly.

**Figure 2:**
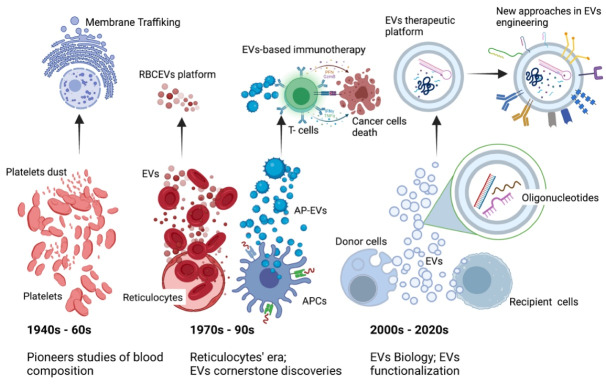
Extracellular vesicles on the move. Experimental observations regarding the EVs biology positively impacted the current success of the EVs based therapeutic platforms. Insights on EVs biogenesis and their role as parental cells “fingerprints” prepared the way for the development of EVs based therapy including immunotherapy and regenerative medicine. Given the EVs role as shuttle of functional molecules, the last decade has documented the flourishment of an increasing number of EVs based drug delivery systems technologies. To date, a series of innovative surface functionalization strategies allow for the improvement of EVs targeting activity, stealth properties and prolonged circulation time. APCs; antigen presenting cells, EVs; extracellular vesicles, AP-EVs; antigen presenting-extracellular vesicles, RBCEVs; red blood cells derived extracellular vesicles

 This section of the review intended to provide a summary of some of the most iconic findings of EVs researchers. We described how experimental observations detailing EVs biology served as the foundation for the recent advancements that are facilitating the translation of EVs-based therapeutics to the clinic (Figure 2). Our group and others have recently published exhaustive review articles that suggest novel strategies to transform naïve EVs into smart drug delivery systems with improved evasiveness and targeting specificity.^6,16,50^ Therefore, descriptions of EVs functional strategies have been omitted to avoid redundancy.

## Recent progresses of EVs as drug delivery system

 As discussed above, the continuous findings of the EVs research field have facilitated the development of various therapeutic platforms, with drug delivery systems (DDS) comprising the most explored use cases. In fact, EVs possess a series of intrinsic features, including biocompatibility, tissue-homing and natural ability of transporting functional molecules. Additionally, EVs offer several previously unexplored possibilities for drug loading and surface engineering which are absent in synthetic DDS.^6^ This section aims to review the recent progress of DDS based EVs with a major emphasis placed on studies that harnessed the innate characteristics of EVs to overcome the current limitation of competitive DDS.

 Delivery of cargo content to its intended site poses a significant challenge for DDS. In this regard, the tetraspanins (Tspan) transmembrane protein family confers EVs with specific organotrophic properties, with Tspan-8 and CD63 driving EVs toward pancreas and neurons, respectively.^51,52^ EVs homing activity has been recently exploited by Hajipour et al^53^ The research group utilized uterine fluid derived exosomes to deliver human chorionic gonadotropin to a difficult target tissue such as the endometrium. Additionally, Zhao et al^54^ harnessed the lung homing ability of exosomes from autologous breast cancer cells to generate a hybrid nanoparticle consisting of albumin and siS100A4 coated with EVs membrane proteins. This hybrid system suppressed post-operative breast cancer metastasis via delivery of siRNA therapeutics.

 Considering that the clearance by leucocytes largely accumulates EVs in the liver, the prospect of using of EVs for the treatment of hepatic diseases possesses great potential. In this respect, Zhang et al^55^ designed a study to investigate the therapeutic potential of liver homing RBCEVs. The group loaded RBCEVs with conventional chemo drugs (Doxorubicin or Sorafenib) or oligonucleotide therapeutics (miR-155-ASOs) and found that they were able to prevent acute liver failure and decrease the proliferation of liver cancer cells both *in vitro* and *in vivo.* On the other hand, when targeting other tissues, macrophage phagocytosis decreases the EVs half-life and negatively impacts the therapeutic efficacy: the correct source of EVs may provide an answer to this problem. In fact, since EVs mirror the properties of parental cells, the use of monocyte and macrophage derived EVs is a reasonable approach to avoid phagocytosis. An interesting study adopted this strategy to extend circulation time and to efficiently deliver antioxidant catalase into neuronal cells. This approach was successful in preventing the exacerbation of Parkinson’s disease both in *in vitro* and *in vivo* models.^56^ However, the ability to cross the blood-brain-barrier (BBB) is confined to small EVs ( < 80 nm in size). In this regard, Yang et al^57^ obtained a high yield of exosomes containing RNA therapeutics and targeting peptides. The group found that the isolated exosomes were able to cross the BBB and restore tumour suppressor function in PTEN-deficient brain glioma animal models.

 EVs are an ideal vector for the *in vivo* delivery of nucleic acid therapeutics because the cargo is shielded from enzymatic degradation. Currently, there are many research articles that have validated the feasibility of EVs based DDS in gene therapy using various disease models.^58^ Of note, Jayasinghe et al^59^ designed RBCEVs functionalized with single domain antibodies to effectively deliver miR-125b to tumors using a metastatic murine breast cancer model. Their research findings showed that use of the functionalized RBCEVs resulted increased silencing of the targeted oncogenic microRNA and a decreased rate of metastasis. An interesting advance in the field of DDS demonstrated that heart-targeting EVs could co-deliver natural molecules, such as curcumin and oligonucleotide therapeutic, to obtain greater cardio protective effects in *in vivo* models of myocardial infarction.^60^ We believe that the co-delivery of different therapeutics is an intriguing strategy that will be further explored in near future.

## EVs: the trigger of the biotechnology market

 EVs research has flourished over the last decade. To date, naïve or engineered EVs are potentially amenable for therapeutic and diagnostic purposes across many research areas, with the fields of oncology and regenerative medicine as major beneficiaries.

 The therapeutic potential of EVs has not gone unnoticed. According to several market analysis reports, the EVs market registered a revenue of approximately $170 million in 2020 and is expected to double by 2026.^61^ Significant growth for the market is expected as EVs will become integrated into the fields of liquid biopsy, precision medicine, and regenerative medicine.^62–64^ Moreover, COVID-19 is a crucial factor that has driven the EVs market over the last two years. The sudden and rapidly expanding outbreak has highlighted the limitations of conventional treatments and had a negative impact on biotech and pharma third-party service providers as well as clinical trials, resulting in trial delay, suspension, or unexpected termination.

 These issues have increased the need for safe, effective, and flexible therapeutics - capable of rapid, on-demand production and optimization - to treat or prevent current or future viral infections. Under these conditions, EVs research steals the show yet again. Indeed, an enormous body of research and review articles pertaining to EVs have been published over the past 2 years. While some groups have investigated the contribution of EVs to viral infection,^42,65–68^ the majority have proposed feasible EVs-based strategies for the diagnosis, prevention, or treatment to reduce viral progression.^69–75^ Of note, with respect to COVID-19, a handful of EVs-based therapies are currently under clinical evaluation (NCT04657458, NCT04276987). The approval of one of these candidates will further reinforce commercial power of EVs.

 Biopharmaceutical companies face intense pressure to bring new therapies to market. In this respect, EVs based technology is an appealing opportunity. Big Pharma are forming strategic collaborations with small or medium size EVs biotech companies to accelerate the development and commercialisation of engineered EVs as a means of delivering therapeutic molecules to treat different pathologies (Table 1).

**Table 1:** Recent Multimillionaire deals involving Big Pharma and EVs biotech

**Companies**	**Details**	**Platform**	**Diseases**	**References**
Jazz Pharmaceuticals; Codiak BioSciences	Codiak obtains US$56 million upfront and up to US$ 200 million for each target	EngEx^TM^	Hematological malignancies and solid tumors	^76^
Sarepta Therapeutic; Codiak BioSciences	Codiak receives US$7 2.5 million upfront and license payments, research funding	EngEx^TM^	Neuromuscular diseases	^77^
Takeda Pharmaceutical Company Limited; Evox	Evox receives US$ 46 million in upfront, the deal worth US$ 880	DeliverEX^TM^	Niemann-Pick disease type C and other undisclosed rare diseases	^78^
Takeda Pharmaceutical Company Limited; Carmine Therapeutics	Carmine signs US$ 900 million research agreement; upfront unrevealed	REGENT^TM^	Undisclosed rare diseases	^79^

 In 2019, Jazz Pharmaceuticals signed an agreement with Codiak BioSciences, a biotech at the forefront of advancing engineered EVs, which could bring in up to $ 1 billion in milestone payments. Codiak received $ 56 million upfront and is slated to receive up to $ 200 million in milestones for each target achieved. In this deal, Codiak will develop pre-clinical and early clinical EVs-based therapeutic platforms directed against previously undruggable targets, including the oncogenes NRAS and STAT3.^76^ Recently, Codiak Therapeutic also signed a two-year agreement with Sarepta Therapeutic, a leader in precision genetic medicine for rare diseases. Codiak is responsible for the design and development of EVs therapeutics to deliver RNA technologies regulating the expression of 5 different genes involved in rare neuromuscular diseases. Accordingly, Codiak will receive up to $ 72.5 million in upfront and near-term license payments plus research funding for each of the targets.^77^ Similarly, a multi-million dollar deal has been signed between Takeda Pharmaceutical Company, a recognised global leader in rare diseases treatment, and Evox. In this multi-target collaboration, Evox’s EVs-based technology is being tailored to deliver protein replacement and mRNA therapies against the Niemann-Pick disease type C and other undisclosed rare diseases.^78^ In the same year, Takeda agreed to collaborate with Carmine Therapeutics. Carmine is a Boston- and Singapore-based biotech company that is dedicated to pioneering innovative therapies based on RBCEVs. Given the absence of nuclear DNA, RBCEVs are naturally designed for safe delivery of oligonucleotide therapeutics. In this respect, Takeda will utilize the Carmine platform to develop treatments against two rare diseases. The deal between the two parties is estimated at $ 900M in total milestone payments plus tiered royalties.^79^

 Other EVs Biotech companies, including ReNeuron and ArunA, have cultivated important partnerships that intend to exploit the ability of certain EVs to cross the BBB with the goal of delivering therapeutics able to target genes involved in the onset or exacerbation of several neurodegenerative diseases.^80^ The details of these deals have not been disclosed.

## EVs in clinical trials: current and future players

 The final goal for any new therapeutic approach is to be directly utilised in humans. In this regard, an increasing number of pharmaceutical companies and start-ups are pivoting their efforts toward translating the EVs pipeline to the clinical setting. EVs, either in their naïve form or functionalized to various extents, are continuously proposed as solutions for unmet biomedical needs.

 However, to date, only a small number of pharmaceutical companies have translated their EVs-based platforms into human clinical trials (Table 2). Indeed, the lack of toxicity and immunogenic responses, as well as the feasibility for a large-scale production, are equally important features to successfully progress EVs-based platforms towards clinical use. As follows, we describe the three classes of EVs that, from our point of view, meet many of the requirements for clinical use.

**Table 2:** Current or completed clinical trials involving MSCEVs or DExs

**Study title**	**Disease**	**Status**	**Evs**	**References**
Effect of Microvesicles and Exosomes Therapy on β-cell Mass in Type I Diabetes Mellitus (T1DM)	Diabetes Mellitus Type 1	Unknown	MSCEVs	NCT02138331
MSC-Exos Promote Healing of MHs	Macular holes	Active, not recruiting	MSCEVs	NCT 03437759
Allogenic Mesenchymal Stem Cell Derived Exosome in Patients With Acute Ischemic Stroke	Cerebrovascular Disorders	Recruiting	MSCEVs	NCT03384433
iExosomes in Treating Participants With Metastatic Pancreas Cancer With KrasG12D Mutation	Pancreatic adenocarcinoma	Recruiting	MSCEVs	NCT03608631
Safety and Efficiency of Method of Exosome Inhalation in COVID-19 Associated Pneumonia	COVID-19	Enrolling by invitation	MSCEVs	NCT04276987
A Global Expanded Access Protocol on Bone Marrow Mesenchymal Stem Cell Derived Extracellular Vesicle Infusion Treatment for Patients With COVID-19 Associated ARDS	COVID-19 ARDS, hypoxia, cytokine storm	Recruiting	MSCEVs	NCT04657458
Vaccination of metastatic melanoma patients with autologous dendritic cell (DC) derived-exosomes	metastatic melanoma	Phase 1 completed	DExs	^81^
Trial of a Vaccination With Tumor Antigen-loaded Dendritic Cell-derived Exosomes	Non Small Cell Lung Cancer	Phase 2 completed	DExs	NCT01159288

###  Mesenchymal stem cell derived EVs

 Mesenchymal stem cells (MSCs) have been explored as a promising therapy in regenerative medicine and immune-related diseases since 1959 when Georges Mathé realized the first ever bone marrow transplant.^82^ With over 1000 clinical trials, MSCs comprise of the most clinically studied experimental cell therapy platform worldwide.^83^ The transplant of stem cells stimulates the regenerative function of adult stem cells located in injured tissue via direct cell-to-cell crosstalk or via secreting factors such as cytokines, growth factors and EVs.^84^ To date, MSCEVs have emerged beyond whole cell-based therapy due to a series of benefits, including a high trans‐differentiation capacity, low structural complexity, no-risk of allogenic immune rejection, and a set of endogenous and exogenous functionalization strategies unsuitable in parental cells. These properties, together with encouraging preclinical data, have enabled researchers to investigate the immuno-modulatory and regenerative properties of MSCEVs in several clinical trials (Table 2). As shown in preclinical studies, the immunosuppressive effect of MSCEVs ameliorated the outcome of type 1 diabetes and autoimmune uveoretinitis *in vivo*.^85^

 These encouraging preclinical data resulted in the design of an ongoing clinical trial testing the effect of repeated doses of MSCEVs in 20 patients with type 1 diabetes (NCT02138331). A second clinical trial based at the Tianjin Medical University began in 2017 (NCT03437759). This trial aims to assess the safety profile and ability of cord tissue derived EVs to promote the healing of large and refractory macular holes. In a different preclinical study, bone marrow-derived EVs engineered with miR-124 facilitated the recovery from brain injury, promoted neurovascular activity after stroke, and prevented post ischemic immunosuppression in mice.^86^ The neurovascular regenerative effect of EVs derived from the same source is now under evaluation in 124 patients affected by acute ischemic stroke (NCT03384433). A fourth clinical trial started in 2018 at M.D. Anderson Cancer Center (NCT03608631). This trial, now actively recruiting, will explore the side effects and optimal treatment dose of MSCEVs loaded with siRNA targeting KrasG12D in metastatic pancreatic ductal adenocarcinoma patients harbouring this specific mutation. As previously reported, MSCEVs suppressed the pro-inflammatory cytokine cascade and protected against oxidative stress in inflammatory lung disease models.^87^ In this regard, an ongoing clinical trial is exploring the efficiency and safety profile of MSCEVs as a treatment to alleviate pneumonia and acute respiratory distress syndrome associated with COVID-19 infection (NCT04276987)

 The therapeutic potency of MCSEVs relies upon the functional protein and RNA cargo which are transferred into recipient cell. Of note, the tissue of origin of the parental cells significantly impacts the content, mechanism of action, and therapeutic efficiency of MSCEVs.^88,89^ Witwer et al^90^ mapped a list of key parameters defining the MSCs and the potential applicability of derived EVs for therapy.

 Currently, MSCEVs-based therapy is hampered by two main obstacles: scalability and excessive cost of production.

 To circumvent these difficulties, pre-conditioning with cytokines, hypoxia, chemical compounds, or genetic manipulation of parental MCSs are well-validated strategies to alter both the content and production of MSCEVs. In a pioneering study, Chen et al immortalized MSCs though the manipulation of MYC to ensure an infinite cellular source of EVs in the milligram range and at low production cost.^91^ Recently, Adlerz et al^92^ proposed a 3D bioreactor system to optimize MSCEVs production in a simple and affordable manner. A different study has demonstrated that extrusion also promotes increased EVs yields. In this case, MCSs pre-treated with paclitaxel (PTX) were extruded using filters with varying pore sizes to obtain a high amount of ultra-pure exosome mimetic loaded PTX.^93^

 In summary, although MSCEVs exhibit a superior safety profile and versatility over their cellular counterparts, various technical obstacles still impede the confirmation of MSCEVs as therapeutics. Most of these limitations are related to the specific cellular source, the lack of a consistent supply of MSCs with a stable phenotype, high costs, and time needed for MSCEVs generation.

 It is thereby critical to overcome these obstacles to realize the clinical utility of MSCEVs-based therapeutics.

###  Dendritic cell derived EVs

 Dendritic cells (DCs) are the orchestrators of the immune response. Acting as the sentinels of the immune system, DCs play an active role in the defence against the infection and contribute to anti-tumor immunity. Currently, the clinical translation of DC-based immunotherapy remains challenging due to the prohibitively high cost and difficulty of preserving DC properties over long time periods.

 DC-derived exosomes (DExs), or DExsosome, provide an attractive alternative for overcoming the technical limitations mentioned above.^94^ DExs possess an intense immunostimulatory activity and a membrane rich in lipids that allows for a longer term storage.^95^

 An early study by Zitvogel et al^96^ demonstrated that the application of DExs as a cell-free vaccine activated T cell response and consequently suppressed tumor growth in a preclinical mastocitoma and carcinoma mouse model. As a result of this pioneering study, the immuno-therapeutic properties of DExs have been confirmed in many preclinical studies and explored in several clinical trials (Table 2).

 A phase I clinical trial evaluated the efficiency of MAGE (Melanoma Associated Antigen) peptide-coated DExs to induce a T cell response in melanoma patients.^81^ A phase II clinical trial used second-generation DExs in advanced NSCLC patients (NCT01159288). This clinical trial investigated the benefit of IFN-γ–DExs as an adjuvant to ameliorate the rate of progression-free survival (PFS) of NSCLC patients previously treated with first-line platinum-based chemotherapy. The disease was stabilized after multiple injections with IFN-γ–DExs in seven out of twenty-two patients (32%), far from the primary goal of the trial (PFS 50%).

 Several considerations need to be mentioned to explain these disappointing results.

 Firstly, the function of DExs may vary according to the biological status of the parental cells. While immature DCs produce DExs with clear immune-stimulatory action, mature DCs generate DExs with immunosuppressive and immune-stimulatory properties.^97^ The unpredictable dynamics that affect the maturation of DCs *in vivo *make it difficult to generate a population of DExs with similar properties. Secondly, the route of administration significantly affects the pharmacokinetic and pharmacodynamic of the DExs. To explain the discrepancy between preclinical and clinical studies, it is important to consider that most preclinical studies administer DExs via intravenous (IV) route to ensure direct entry of the EVs into the bloodstream. On the contrary, unless strictly necessary, clinical settings tend to avoid invasive IV administration and instead opt for subcutaneous injection.

 Finally, eligible patients may likely have been recipients of other therapeutic regimens before participating in the trials, thereby impacting the immune system and hence the outcome of DExs treatment.

 Overall, there is great promise in DExs based immunotherapy. Future advances in bioengineering will surely help to overcome the limitations of this platform.

###  Red blood cell derived EVs

 Red blood cells (RBCs) -the most abundant cells in the human body (~83% of the total cells)-^98^ function as transporters of oxygen from lung to peripheral tissues: the absence of a voluminous nucleus allows for increased haemoglobin content and facilitates plasticity when moving through the vasculature. Moreover, increased expression of the anti-phagocytic protein CD47 extends *in vivo* circulation time (~120 and ~50 days for human and mouse RBCs respectively).^99^

 It is worth mentioning that the clinical successes of MSCEVs and Dexs are also hampered by two challenges: (i) the difficulty of producing large amounts of homogeneous EVs, and (ii) the risk of gene transfer associated with the DNA content of EVs. In this respect, the characteristics of RBCs render them ideal EVs source. Indeed, RBCs are readily available from any blood bank and unlike other mammalian cells, RBCs lack both nuclear and mitochondrial DNA, making horizontal oncogene transfer unlikely.

 These properties, together with a safe profile validated over decades of blood transfusions, facilitated the development of an innovative platform based on the functionalization of RBCs derived EVs as delivery vectors of oligonucleotides or small molecule therapeutics.

 An interesting study enriched RBCEVs with exogenous cholesterol to retain a pH gradient that facilitated the loading of the chemotherapeutic doxorubicin or the antibiotic vancomycin.^100^ The findings of the study showed that loaded RBCEVs produced higher therapeutic effect than that of free drug in both orthotopic 4T1 breast cancer and in a methicillin-resistant *Staphylococcus aureus* skin infection murine models.

 As EVs are able to transfer nucleic acids between cells *in vivo*, it seems relatively straightforward their application for the purpose of gene therapy. Our research group investigated the preclinical use of RBCEVs as RNA-based therapeutic carriers. In our pilot study, a variety of RNA-based therapeutics, including antisense oligonucleotide (ASO), Cas9 mRNA, and guide RNAs were electroporated into RBCEVs.^46^ We observed higher efficiency of RNA delivery and lower toxicity compared to commercially available transfection reagents. In terms of therapeutic response, treatment with miR125b ASO loaded RBCEVs decreased cancer cell proliferation and metastasis in breast cancer and leukaemia *in vivo* models. As a follow-up to those findings, we recently extended the application of our platform by validating a method for increasing the targeting specificity of RBCEVs.^101^ Briefly, we used protein sortase or ligase enzymes to covalently link a large number of targeting motifs (peptides, single domain antibody and monoclonal antibody) onto RBCEVs. The functionalization resulted in improved uptake of RBCEVs exclusively by cells expressing the specific targets, such as HER2, SIRP alpha, EGFR or CXCR4 receptors. Furthermore, systemic delivery of engineered RBCEVs enhanced the curative efficacy of different therapeutic cargoes, including PTX, oligonucleotide therapeutics, and pro-apoptotic peptides in *in vivo* mouse model of solid and liquid cancers.^59,101^

 RBCEVs based therapy has just begun, and thus far, to the best of our knowledge, there are no active clinical trials investigating their therapeutic potential.

 Although the absence of the nucleus precludes RBCs and derived EVs from genetic modification, RBCEVs currently have several advantages that are absent in other EV-based platforms, including extraordinary biosafety and scalability. Thus, we believe that RBCEVs-based therapy will soon be actively investigated in clinical trials.

## Take-home messages for a bright future ahead

 To date, many obstacles hamper the clinical translation of EVs based platform and several questions need to be posed for future studies. Herein, it is fundamental to curb enthusiasm and carefully analyse the current status.

 The vast majority of studies detail the impressive performance and curative potential of EVs, but we need to emphasize that encouraging preclinical data has rarely found equal success in clinical settings.

 Indeed, the lack of standardized protocols for EVs isolation, purification, and functionalization as well differences in EVs source may contribute to significant variation among studies, thereby affecting the reproducibility and reliability of the trial data.^16^ New approaches are needed to ameliorate the quality of EVs isolated and purified from clinical specimens. In this regard, innovative methods such as asymmetric flow field-flow fractionation and acoustic trapping have demonstrated an exciting alternative for the isolation and enrichment of ultrapure EVs subpopulations.^102,103^ Recently, Chen et al^104^ proposed a straightforward dielectrophoretic method to reduce the isolation time to 30 minutes while preserving a high level of recovery and purity in EVs isolated from plasma of lung cancer patients.

 While the aforementioned approaches are undergoing optimization, a combination of well-corroborated isolation techniques, including PEG precipitation plus immune-capture, ultracentrifugation plus SEC or ultrafiltration plus asymmetrical-flow field-flow fractionation,^46,105,106^ may represent an immediate solution. Future research should certainly make use of protocols specifically tailored to isolate of EVs specific human sample types, including blood,^46^ plasma^107^ or urine.^106^

 Of significance, most of EVs research has focused on the application of EVs as delivery vectors of small molecule therapeutics. Despite the enormous efforts made to improve various aspects, such as delivery efficacy, specificity, and safety profile, none of these functionalized EVs have currently transitioned to the clinic. One key reason contributing to this issue is the countless number of challenges faced during the exhausting pre-clinical optimization of the EVs based platforms.^3^ An exciting solution may come from advanced computational frameworks, including artificial intelligence and computational predictive modelling. These computational aids decode an enormous amount of data to obtain predictive hits that could enable more reliable and time-efficient EVs functionalization.^108^

 Additionally, the lack of knowledge with respect to the *in vivo* behaviour of EVs needs to be taken into consideration: *in vivo* toxicity, tissue distribution, and clearance are important factors that require major investigation. In this regard, it is worth of mentioning the study by Montaner-Tarbes et al^109^ Using a porcine model, the authors ascertained the side effects of multiple injections of EVs isolated from the serum of convalescing animals with a prior porcine reproductive and respiratory syndrome virus infection. In an equally relevant study, Potz et al^110^ demonstrated that myocardial injection of MSCEVs increased vessel density and blood flow to ischemic myocardial tissue in a porcine model. These findings endorse the clinical application of MSCEVs in human patients of chronic myocardial ischemia.

 For obvious reasons, most preclinical studies utilize small animal models such as mice, rats or hamsters. Other models, such as pigs or monkeys, which possess anatomy and physiology more similar to humans, would address many of the issues related to EVs pharmacokinetics and pharmacodynamics.

 Despite the aforementioned obstacles, EVs-based therapeutics have gained traction in several human trials and the trend is expected to increase in near future. Most of the studies in clinical trials have harnessed the intrinsic therapeutic potential of naïve EVs as regenerative and immunomodulation agents. However, pharmaceutical companies and start-ups are investing considerable technical efforts and financial resources to develop new classes of engineered EVs-based platforms with the aim of assessing their therapeutic potential in clinical trials.

 Another hot topic of investigation is the use of EVs as biomarkers for the detection of diseases or for monitoring patient treatment response. In this respect, EV-derived small RNAs have been recently proposed for the diagnosis of HIV infection,^111^ neurodegenerative diseases,^112^ cancers.^113^ Moreover, several reports adopted EVs as biomarkers for the early detection of less common life-threatening diseases, such as preeclampsia or other reproductive complications.^114,115^ However, the difficulty to obtain large biofluid volumes may impede the reproducibility of those data and consequently, even if promising, hamper the clinical applicability of these types of EVs biomarkers.

 In summary, EVs technology is an innovative and highly promising platform of potent cell-free therapies that may address a wide set of human conditions.

## Conclusion

 The discovery of EVs as intercellular messengers is a fundamental breakthrough in cellular biology. As highlighted in this mini-review, the possibility to turn this natural carrier of information into a smart nanotherapy is a real opportunity. There is no doubt that EVs based technology will make a positive impact in human healthcare; however, this new era of technology has just begun.

 The EVs field is moving quickly from the lab bench to the clinical setting and is likely to contribute significantly to human health.

## Acknowledgments

 Figure was generated using BioRender.com. Final editing and proofreading

 were performed by Dr. Kyle Vaughn Laster.

## Competing Interests

 The author declares that he has no competing interests.

## Ethical Approval

 Not applicable.
